# Iso-α-Acids, Bitter Components in Beer, Suppress Inflammatory Responses and Attenuate Neural Hyperactivation in the Hippocampus

**DOI:** 10.3389/fphar.2019.00081

**Published:** 2019-02-11

**Authors:** Yasuhisa Ano, Misato Yoshikawa, Yuta Takaichi, Makoto Michikawa, Kazuyuki Uchida, Hiroyuki Nakayama, Akihiko Takashima

**Affiliations:** ^1^Graduate School of Agricultural and Life Sciences, The University of Tokyo, Tokyo, Japan; ^2^Research Laboratories for Health Science & Food Technologies, Kirin Company Ltd., Kanagawa, Japan; ^3^Department of Aging Neurobiology, National Center for Geriatrics and Gerontology, Obu, Japan; ^4^Department of Biochemistry, School of Medicine, Nagoya City University, Nagoya, Japan; ^5^Faculty of Science, Gakushuin University, Tokyo, Japan

**Keywords:** Alzheimer’s disease, amyloid β, cognitive decline, hippocampus, inflammation, iso-α-acids, lipopolysaccharide

## Abstract

Due to the growth in aging populations worldwide, prevention and therapy for age-related cognitive decline and dementia are in great demand. We previously demonstrated that long-term intake of iso-α-acids, which are hop-derived bitter compounds found in beer, prevent Alzheimer’s pathology in a rodent model. On the other hand, the effects of iso-α-acids on neural activity in Alzheimer’s disease model mice have not been investigated. Here, we demonstrated that short-term intake of iso-α-acids suppresses inflammation in the hippocampus and improves memory impairment even after disease onset. Importantly, we demonstrated that short-term administration of iso-α-acids attenuated the neural hyperactivation in hippocampus. In 6-month-old 5 × FAD mice exhibiting hippocampus inflammation and memory impairment, oral administration of iso-α-acids for 7 days reduced inflammatory cytokines, including MIP-1α and soluble Aβ and improved object memory in the novel object recognition test. In 12-month-old J20 mice, intake of iso-α-acids for 7 days also suppressed inflammatory cytokines and soluble Aβ in the brain. Manganese-enhanced magnetic resonance imaging (MEMRI) of hippocampi of J20 mice showed increased manganese compared with wild type mice, but iso-α-acids canceled this increased MEMRI signal in J20 mice, particularly in the hippocampus CA1 and CA3 region. Taken together, these findings suggest that short-term intake of iso-α-acids can suppress hippocampus inflammation even after disease onset and improve hyper neural activity in Alzheimer’s disease model mice.

## Introduction

The rise in aging populations worldwide is accompanied by increasing rates of dementia and cognitive impairment, which are a burden to national healthcare systems as well as patients and their families. Because of the lack of treatments for dementia, preventive approaches such as diet, exercise, and lifelong learning have received increasing research attention. In etiological studies of lifestyle, low to moderate consumption of alcoholic beverages, such as wine and beer, might reduce the risk of cognitive decline and the development of dementia. Individuals who consume low to moderate levels of alcohol on a daily basis were shown to have significantly lower risks of neurodegenerative diseases compared with individuals who abstained from alcohol or drank heavily (Matsui et al., 2011; Neafsey and Collins, 2011; Horvat et al., 2015). Apart from the effects of alcohol itself, resveratrol, a polyphenolic compound found in red wine, has been shown to have neuroprotective effects (Vidavalur et al., 2006; Arntzen et al., 2010; Porquet et al., 2014; Witte et al., 2014). Our group previously demonstrated that long-term intake of iso-α-acids for 3 months, which are bitter components in beer, prevented Alzheimer’s pathology in a transgenic mouse model. Iso-α-acids are derived from hops, the female inflorescences of the hop plant (*Humulus lupulus* L.), and have been used in beer production since 822. Hops are used as both a preservative and a flavoring agent in the beer-brewing process. Iso-α-acids were shown to activate the peroxisome proliferator-activated receptor-γ (PPAR-γ) and regulate microglial phagocytosis and inflammation (Ano et al., 2017). Our group has previously demonstrated that iso-α-acids prevented dyslipidemia and type 2 diabetes in a diet-induced obese rodent model (Yajima et al., 2004, 2005), and improved glucose metabolism and decreased body fat in a clinical trial (Obara et al., 2009). Long-term administration of iso-α-acids is applicable for the preventive approaches, but the effects for the therapeutic approaches and for neural activity have not been elucidated. It is reported that hyperactivity in hippocampus is associated with cognitive impairment and improvement of the hyperactivity show therapeutic effects on memory impairment (Bakker et al., 2012). In addition, previous study did not conclude that long-term administration of iso-α-acids suppressed the inflammation in the brain directly or as a result of the improvement of amyloid β (Aβ) deposition. To address these research gaps, in the present study, we examined the effects of short-term intake of iso-α-acids on brain inflammation and neural activity in hippocampus using the manganese-enhanced magnetic resonance imaging (MEMRI) in Alzheimer’s model mice.

## Materials and Methods

### Animals

Alzheimer’s disease model, B6SJL-Tg mice [APPSwFlLon, PSEN1^∗^M146L^∗^L286V^1^, (Oakley et al., 2006)], hereafter referred to as 5 × FAD transgenic mice, were purchased from Jackson Laboratory (Sacramento, CA, United States) and maintained by crossing hemizygous transgenic mice with B6SJLF1/J mice at the experimental facility of the University of Tokyo. The 5 × FAD transgenic mice overexpress mutant human APP (695) with the Swedish (K670N, M671L), Florida (I716V), and London (V717I) Familial Alzheimer’s Disease (FAD) mutations, along with human PS1 harboring two FAD mutations, namely, M146L and L286V. Non-transgenic wild type (WT) littermates as controls were used in the experiments. All experiments were approved by the Animal Care and Use Committee of the Graduate School of Agricultural and Life Sciences, The University of Tokyo, and conducted in strict accordance with its guidelines. Transgenic (J20) mice express human amyloid precursor protein (hAPP) with the Swedish (K670N, M671L) and Indiana (V717F) mutations under the control of the PDGF β-chain promoter (Mucke et al., 2000). Experiments using J20 mice were approved by the local ethical board and complied with the guidelines for animal experimentation of the National Center for Geriatrics and Gerontology in Japan. Pregnant C57BL/6J mice and 6-week-old CD-1 (ICR) mice were purchased from Charles River Japan (Tokyo, Japan) and maintained at a faculty of Kirin Company Ltd. The experiments were approved by the Animal Experiment Committee of Kirin Company Ltd. and conducted in strict accordance with its guidelines since 2014–2016. All efforts were made to minimize animal suffering. Mice were fed a standard rodent diet (CE-2, CLEA Japan, Tokyo, Japan) and maintained at room temperature (23 ± 1°C) under a constant 12-h light/dark cycle (light period from 8 a.m. to 8 p.m.).

### Preparation of Iso-α-Acids

α-Acids predominantly consist of three congeners: cohumulone, humulone, and adhumulone. During the brewing process, they are each isomerized into two epimeric isomers, namely, cis- and trans-iso-α-acids. Purchased isomerized hop extract (IHE) (Hopsteiner, Mainburg, Germany) with 30.5% (w/v) iso-α-acids, comprising trans-isocohumulone (1.74% w/v), cis-isocohumulone (7.61% w/v), trans-isohumulone (3.05% w/v), cis-isohumulone (14.0% w/v), trans-isoadhumulone (0.737% w/v), and cis-isoadhumulone (3.37% w/v) as described previously (Taniguchi et al., 2013).

### Primary Microglia Cell Culture

Microglial cells were isolated from brains of newborn C57BL/6J mice (<7 days old) via magnetic cell sorting (MACS) after conjugation with anti-CD11b antibody, as described previously (Ano et al., 2015). Briefly, isolated CD11b-positive cells (>90% pure, evaluated by flow cytometer) were plated into poly-D-lysine (PDL)–coated 96-well plates (BD Biosciences, Billerica, MA, United States) and cultured in a DMEM/F-12 (Gibco, Carlsbad, CA, United States) medium supplemented with 10% fetal calf serum (Gibco) and 100 U/ml penicillium/streptomycin (Sigma-Aldrich, St. Louis, MO, United States). Microglia at a density of 30,000 were treated with each iso-α-acids for 12 h, and then with lipopolysaccharide (LPS, 5 ng/ml, Sigma-Aldrich, St. Louis, MO, United States) and interferon-γ (IFN-γ, 0.5 ng/ml, R&D systems, Minneapolis, MN, United States) for 12 h. After stimulation, the supernatant was used for the TNF-α production assay. Concentrations of cytokines sand chemokines in the supernatant was measured by the Bio-Plex assay system (Bio-Rad, Hercules, CA, United States).

### Neuronal Inflammation Induced by Lipopolysaccharide

Six-week-old ICR male mice were orally administered 1 mg/kg of iso-α-acids dissolved in distilled water once a day for 3 days. One hour after the final administration, the mice were deeply anesthetized with sodium pentobarbital (Kyoritsu Seiyaku, Tokyo, Japan) and injected intracerebroventricularly with 0.25 mg/kg of LPS (5 μl, L7895, Sigma) or PBS as controls as previously described (Ano et al., 2015). Briefly, a micro syringe with a 27-gauge stainless steel needle, 2 mm in length, was used for micro injection. The needle was inserted unilaterally 1 mm to the right of the midline at the equal distance between eyes and ears, and perpendicular to the plane of the skull (anteroposterior, -0.22 mm from the bregma; lateral, 1 mm from the bregma). LPS was delivered gradually within 30 s. The needle was withdrawn after waiting 30 s. Twenty-four hour later, the mice were subjected to the Y-maze test for 8 min to evaluate spontaneous alternation. After the test, the hippocampus and cerebral cortex were removed and homogenized in TBS buffer containing a protease inhibitor cocktail (BioVision, CA, United States). To quantify cytokine and chemokine production, the homogenate supernatant was measured by the Bio-Plex assay system (Bio-Rad).

### Aβ and Cytokine Measurement in Transgenic Mice

To evaluate the effects of iso-α-acids on Alzheimer’s-like disease, 6-month-old transgenic 5 × FAD and WT mice were orally administered daily 1 mg/kg iso-α-acids for 7 days. The mice were subjected to a novel object recognition test after 7 days of administration, and their brains were then removed for quantification of Aβ and cytokines. The hippocampus and cerebral cortex were homogenized in TBS buffer (Wako) with a multibeads shocker (Yasui Kikai, Osaka, Japan). After centrifugation at 50,000 × *g* for 20 min, the supernatant was collected. Pellets were re-homogenized in TBS containing 1% Triton X-100 (Wako) and the supernatant was collected after centrifugation. The total protein concentration of the supernatant was measured with the BCA protein assay kit (ThermoScientific, Yokohama, Japan). The first supernatant was assayed for quantification of soluble Aβ1-42 (Wako) by enzyme-linked immunosorbent assay (ELISA). To quantify cytokines and chemokines, supernatants were evaluated by the Bio-Plex assay system (Bio-Rad). The second supernatant was used for quantification of insoluble Aβ1-42 (Wako) by ELISA.

### Spontaneous Alternation Test

The spontaneous alternation test was performed as previously described (Ano et al., 2018). The test used a three-arm Y-maze with equal angles between all arms (25 cm long × 5 cm wide × 20 cm high) and with walls constructed from dark black polyvinyl plastic. Each mouse was initially placed in one arm, and the sequence and number of arm entries were counted for 8 min. The alternation score (%) for each mouse was defined as the ratio of the actual number of alternations to the possible number (defined as the total number of arm entries minus two) multiplied by 100, i.e., % Alternation = [(Number of alternations)/(Total arm entries-2)] × 100.

### Novel Object Recognition Test

An object recognition test was performed during the light period in a polyvinyl chloride box (25 cm × 40 cm × 20 cm) without a roof, in accordance with previous work (Ayabe et al., 2018). For the acquisition trial, a pair of wooden triangle poles (4.5 cm × 4.5 cm × 4.5 cm) or wooden pyramids (4.5 cm × 4.5 cm × 4.5 cm) was used; for the retention trial, a pair of poles or pyramids and a golf ball (4.5 cm diameter) were used. In all trials, the objects were placed 7.5 cm from the corner of the box. In the acquisition trial, 1 h after oral administration of the test sample, the mouse was allowed to explore the box containing the two objects for 10 min. Twenty-four hour later, 1 h after administration of the test sample, the mouse was allowed to explore the box containing the novel and familiar objects for 5 min. The discrimination index (DI) was calculated by dividing the difference in time exploring the novel object and familiar object by the total time spent exploring both objects, i.e., (novel object exploration time - familiar object exploration time)/(total exploration time); thus, a DI of 0 indicated equal exploration of both objects.

### Manganese-Enhanced MRI

Manganese-enhanced magnetic resonance imaging was performed in accordance with our previous report (Yoshikawa et al., 2018). Mice were administered with MnCl_2_ (20 mg/kg i.p.) and then returned to their home cages after 30 min, they were exposed to a novel environment (clear Perspex cylinder, 30-cm diameter) for 2 h (cylinder moved every 30 min to prevent habituation) and they were video-recorded. The mice were then returned to their home cage for 90 min before MRI scanning.

Anesthesia was induced with 3.0% isoflurane/air and maintained with 0.5–1.5% isoflurane/air; and deep core body temperature and heart rate were monitored (SA Instruments, Inc., United States) throughout the procedure. Scanning was performed 4 h after MnCl_2_ injection using a 4.7T AVANCE III PharmaScan (Bruker BioSpin, Germany). Radio frequency transmission and reception were applied with a 23 mm inner diameter birdcage volume coil. Images were acquired with 3D Fast Imaging using a Steady-State Free Precession (FISP) sequence [repetition time (TR) = 8 ms, echo time (TE) = 4 ms, flip angle = 20°, number of acquisition = 7, matrix = 160 × 160 × 160, field of view (FOV) = 20 mm × 20 mm × 20 mm, and voxel size = 0.125 mm × 0.125 mm × 0.125 mm]. The total acquisition time was 31 min. MRI data were analyzed as described previously (Kimura et al., 2007), with the aid of a custom-developed MATLAB function (2012a, MathWorks). Brain slices were aligned with reference to the bregma. MR images were realigned and registered non-rigidly to the mouse brain template constructed by aligning and averaging 10 subject images. All voxel data were smoothed using a 3D Gaussian filter (MATLAB Image Processing Toolbox, version 5.02, MathWorks). Image intensities were normalized to the mean signal in the whole brain of each individual mouse. MR images were visualized with Osirix (version 5.0.2), an open-source software for navigating multidimensional DICOM images.

### Statistical Analysis

The data represent the mean and the error bars indicate the SEM. Data were analyzed by Student’s *t*-test and one-way analysis of variance (ANOVA) followed by Dunnett’s test or the Tukey–Kramer test were performed, as indicated in the Figure legends. All statistical analyses were performed using the Ekuseru-Toukei (2012) software program (Social Survey Research Information, Tokyo, Japan). A *p*-value <0.05 was considered statistically significant.

## Results

### Effects of Iso-α-Acids on Microglial Cytokine and Chemokine Productions

**FIGURE 1:**
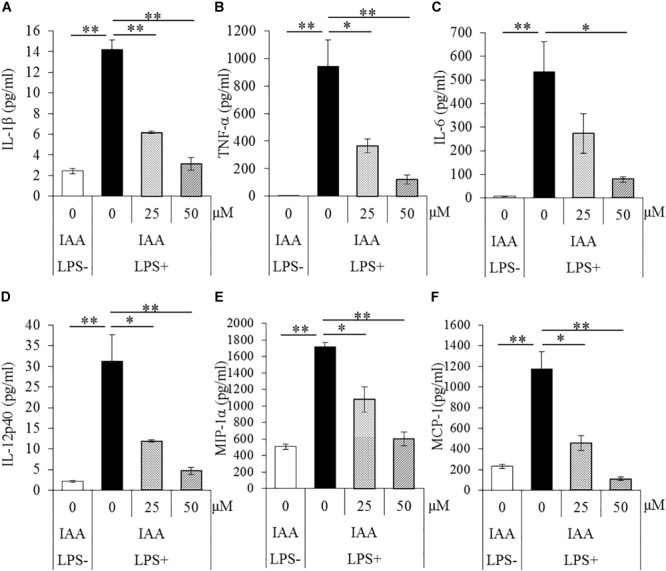
Effects of iso-α-acids on microglial cytokine and chemokine productions. **(A–F)** The amounts of IL-1β, TNF-α, IL-6, IL-12p40, MIP-1α, and MCP-1 in the supernatant of microglial culture pretreated with 0, 25, and 50 μM iso-α-acids and treated with 5 ng/ml LPS and 0.5 ng/ml IFN-γ. IAA: iso-α-acids. Mean ± SE of three wells per sample. The *p*-values shown in the graph were calculated by Student’s *t*-test (LPS [–] vs [+] at 0 μM) and one-way ANOVA followed by Dunnett’s test. ^∗^*p* < 0.05 and ^∗∗^*p* < 0.01.

To evaluate the effects of iso-α-acids on primary microglia culture, cytokines and chemokines in the supernatant of microglial culture treated with LPS were quantified. The concentrations of IL-1β, TNF-α, IL-6, IL-12p40, MIP-1α, and MCP-1 in supernatant were significantly increased after LPS treatment. The concentrations were, however, significantly reduced by iso-α-acids addition concentration-dependently (Figure 1A–F, respectively). These results suggest that iso-α-acids suppress inflammatory cytokines and chemokines.

### Effects of Iso-α-Acids on Cytokine Productions and Memory Impairment in Mice Treated With LPS

**FIGURE 2:**
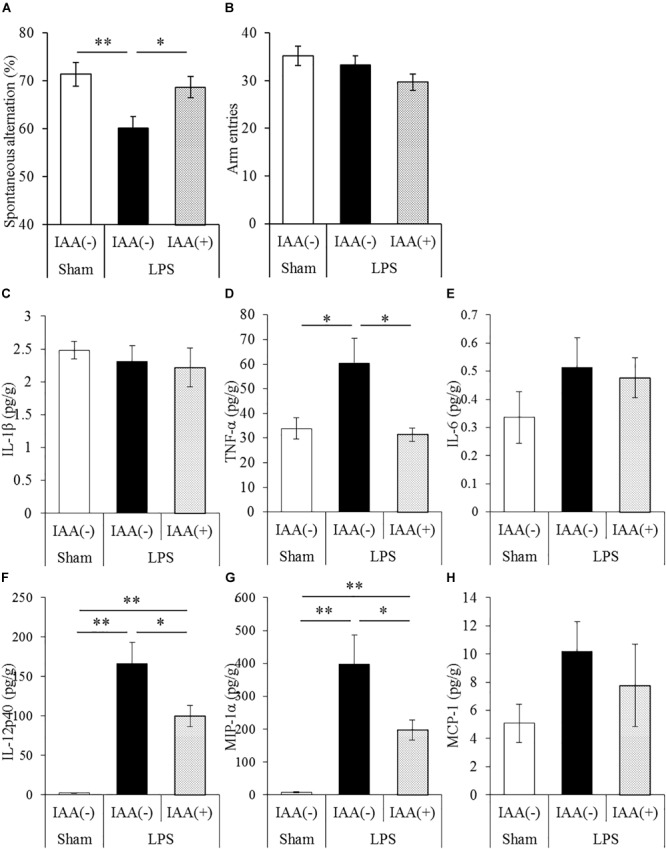
Effects of iso-α-acids on memory impairment induced by LPS. **(A,B)** CD-1 (ICR) mice were orally administered with 0 or 1 mg/kg of iso-α-acids for 3 days and intracerebroventricularly injected with PBS or 0.25 mg/kg LPS 1 h after the last iso-α-acids administration. Mice were subjected to the spontaneous alternation test using the Y-maze 1 day after LPS injection. Spontaneous alternations **(A)** and arm entries **(B)** in the Y-maze test were recorded to evaluate spatial memory. **(C–H)** Concentrations of IL-1β, TNF-α, IL-6, IL-12p40, MIP-1α, and MCP-1 in the hippocampus were measured 24 h after LPS injection, respectively. Data are represented as mean ± SE of 10 mice per group. *p*-Values shown in the graph were calculated by one-way ANOVA followed by the Tukey–Kramer test. ^∗^*p* < 0.05 and ^∗∗^*p* < 0.01.

To evaluate the effects of iso-α-acids on cytokine productions and memory impairment in LPS-injected mice, mice inoculated with LPS were subjected to the Y-maze test. LPS treatment significantly reduced spontaneous alternation, whereas oral administration of iso-α-acids significantly improved the reduction (Figure 2A). There were no significant changes in arm entries among each group (Figure 2B). Next, we measured the levels of cytokines in the hippocampus. Concentrations of TNF-α, IL-12p40, and MIP-1α in the hippocampus of LPS-injected mice were significantly increased compared to those in untreated mice. The concentrations of TNF-α, IL-12p40 and MIP-1α significantly decreased in mice orally administered with iso-α-acids (Figure 2D,F,G). However, the concentrations of IL-1β, IL-6 and MCP-1 were unchanged by LPS injection (Figure 2C,E,H, respectively). These results showed that oral administration of iso-α-acids improved memory impairment and suppressed cytokine productions in LPS-injected mice.

### Effects of Iso-α-Acids on Inflammation and Memory Impairment in 5 × FAD Mice

**FIGURE 3:**
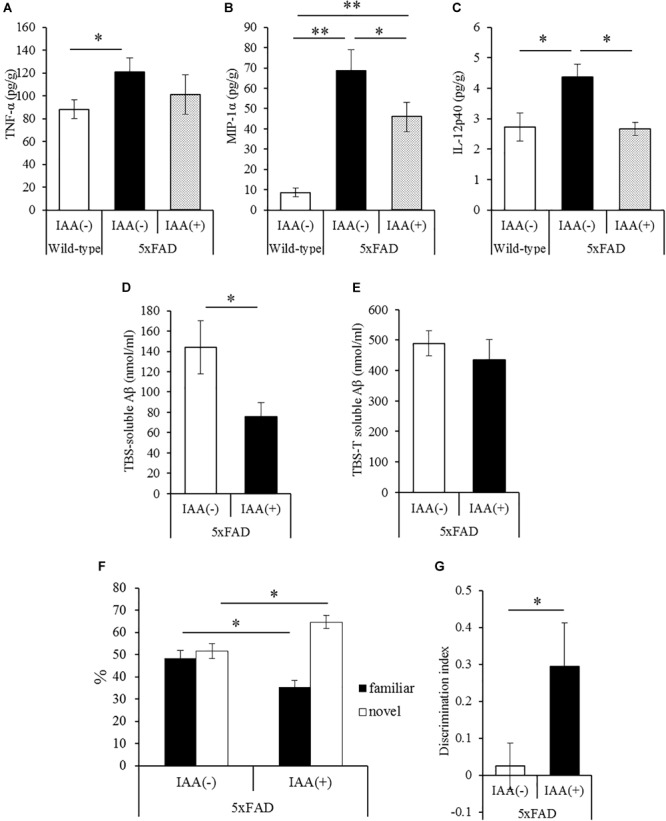
Effects of iso-α-acids on 5 × FAD Alzheimer’s disease model mice after disease onset. **(A,C)**, 5 × FAD mice were orally administered with 0 or 1 mg/kg iso-α-acids for 7 days, and the levels of TNF-α **(A)**, MIP-1α **(B)** and IL-12p40 **(C)** in the hippocampus was measured, respectively. **(D,E)** The levels of TBS soluble **(D)** or TBS insoluble and TBS-T soluble **(E)** Aβ_1-42_ in the hippocampus, respectively. **(F,G)** On days 6 and 7 of administration, 5 × FAD mice were subjected to the novel object recognition test. The time spent exploring novel and familiar objects during 5 min of re-exploration was measured **(F)**. The discrimination index = [time spent with object A – time spent with object B]/[total time exploring both objects] is shown **(G)**. Data are mean ± SE of 10 mice (wild type mice) and 8 mice (5 × FAD mice per group). *p*-Values were calculated by Student’s *t*-test **(D–G)** and one-way ANOVA followed by the Tukey–Kramer test **(A–C)**. ^∗^*p* < 0.05 and ^∗∗^*p* < 0.01.

To evaluate the effects of iso-α-acids on inflammation and cognitive impairment after disease onset, 5 × FAD mice were administered with 1 mg/kg iso-α-acids for 7 days. In 5 × FAD mice, long-term intake of iso-α-acids for 2.5 months resulted in the reduction of Aβ deposition and in inflammation in the brain (Ano et al., 2017). The amounts of MIP-1α and IL-12p40 in 5 × FAD mice administered with iso-α-acids was significantly lower than those in control 5 × FAD mice, and was significantly increased in control 5 × FAD mice relative to WT mice (Figure 3B,C). The amount of TNF-α was also increased in control 5 × FAD mice compared to that in WT mice, while the increase was not observed in 5 × FAD mice treated with iso-α-acids (Figure 3A). The amount of TBS-soluble Aβ in the hippocampus of 5 × FAD mice treated with iso-α-acids was significantly reduced compared to that in control 5 × FAD mice (Figure 3D). The amounts of TBS insoluble and TBS-T soluble Aβ in the hippocampus of 5 × FAD mice treated with iso-α-acids were unchanged (Figure 3E). 5 × FAD mice were also subjected to a novel object recognition test. The mice treated with iso-α-acids explored a novel object for a longer time than the control 5 × FAD mice. The amounts of time spent approaching the novel object and DI of 5 × FAD mice treated with iso-α-acids were significantly higher than that of control 5 × FAD mice (Figure 3F,G). These results suggest that short-term treatments with iso-α-acids suppress the levels of inflammatory cytokines and soluble Aβ in the hippocampus, which lead to improve memory impairment even after the onset of Alzheimer’s pathology.

### Effects of Iso-α-Acids on Inflammation and Neural Activity in J20 Mice

**FIGURE 4:**
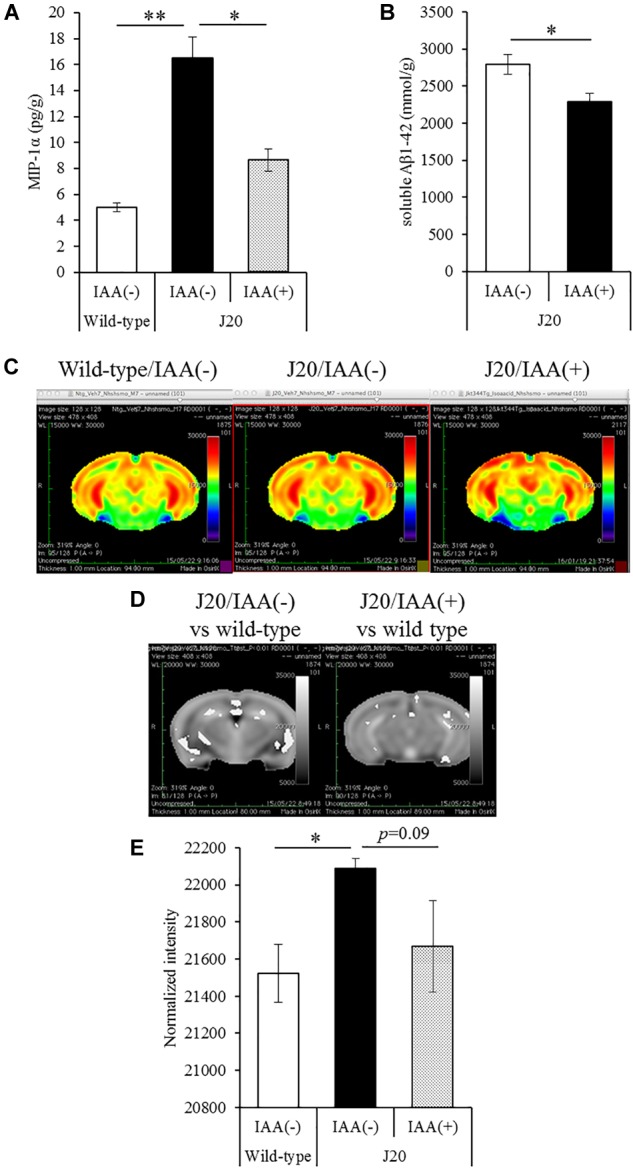
Effects of iso-α-acids on J20 Alzheimer’s disease model mice. **(A,B)** J20 mice were orally administered with 0 or 1 mg/kg iso-α-acids for 7 days. The levels of MIP-1α **(A)** and TBS soluble Aβ_1-42_ in the hippocampus was measured, respectively. **(C)** Coronal brain sections from wild type mice (left), J20 mice without iso-α-acids (middle), and J20 mice treated with iso-α-acids (right), all of which were 12 months old. Relative MRI signal intensities, after normalization to the mean signal intensity in the whole brain, are depicted (see color spectrum scale bar). **(D)** Coronal brain sections showing the differences of signal intensities J20 mice between wild type mice and J20 mice without iso-α-acids (left), and between wild type mice and J20 mice with iso-α-acids (right). **(E)** Normal intensities of hippocampus data are mean ± SE of seven mice (wild type mice), eight mice [IAA(–) J20 mice], and five mice [IAA(+) J20 mice]. *p*-Values shown in the graph were calculated by Student’s *t*-test **(B)** and one-way ANOVA followed by the Tukey–Kramer test **(A,E)**. ^∗^*p* < 0.05 and ^∗∗^*p* < 0.01.

To evaluate the effects of a short-term treatments of iso-α-acids, 12-month-old J20 mice were administered 1 mg/kg iso-α-acids for 7 days and then subjected to MEMRI. The level of MIP-1α in the brain of the mice treated with iso-α-acids was significantly lower than that in control mice, and the level of which was significantly increased compared to that of WT mice (Figure 4A). The level of TBS soluble Aβ in the hippocampus of J20 mice treated with iso-α-acids was significantly reduced compared to control J20 mice (Figure 4B).

To evaluate the neural activity, mice were injected with MnCl_2_, allowed to explore a novel space, and then subjected to MRI to detect manganese. Manganese was distributed in the hippocampus and cerebral cortex (Figure 4C). The deposition of manganese in some areas of the hippocampus, especially the CA1and CA3 region, of control J20 mice was significantly increased compared to that of WT mice, whereas the increase of deposition was attenuated by iso-α-acids treatment (Figure 4D). The normalized intensity of manganese in the whole hippocampus of control J20 mice was significantly increased compared to that of WT mice, whereas an increase in J20 was returned to the level of WT mice by the treatment of iso-α-acids (Figure 4E). These results suggest that iso-α-acids suppress inflammation and improve the neural activity in the hippocampus.

## Discussion

The present study demonstrated that short-term treatment of iso-α-acids as 7 days suppressed inflammation in the hippocampus and improved memory impairment in Alzheimer’s disease model mice, even after disease onset. Notably, short-term administration of iso-α-acids attenuate the neural hyperactivation in hippocampus in Alzheimer’s disease model mice detected by MEMRI. Iso-α-acids treatment suppressed the cytokine and chemokine levels in the hippocampus and improved memory impairment induced by LPS injection. The spontaneous alternation test used in this study evaluates spatial working memory, which is a hippocampus-dependent memory function (Lalonde, 2002). We previously reported that iso-α-acids activate the PPAR-γ (Yajima et al., 2004) and that the PPAR-γ activation is involved in the suppression of microglial inflammation (Ano et al., 2017). It has also been reported that PPAR-γ activation changes microglia phenotype to the M2 anti-inflammatory type (Pan et al., 2015; Wen et al., 2018). Pioglitazone, which is a PPAR-γ agonist, also alters microglia to the M2 type *in vivo* (Mandrekar-Colucci et al., 2012). These results suggest that iso-α-acids suppressed the inflammatory response via activation of PPAR-γ and suppression of microglial inflammation in the hippocampus by iso-α-acids may contribute to the prevention of memory impairment. On the other hand, the phenotype of inflammation induced by LPS, which is toll-like receptor agonist, is different from that in the brain of Alzheimer’s disease.

Inflammation in the brain has attracted growing attention to a preventive and therapeutic strategy for Alzheimer’s disease (Fernandez et al., 2013). Epidemiological investigation has suggested that an intake of non-steroidal anti-inflammatory drugs (NSAIDs) has a preventive effect against Alzheimer’s disease (Breitner et al., 1995; Stewart et al., 1997), suggesting the therapeutic potential of pioglitazone. Microglia are known to play a crucial role in inflammation in the brain, and generally remove old synapses and waste products in the brain to maintain the environment (Kettenmann et al., 2013), yet massively activated microglia produce neurotoxic substances, including reactive oxygen species and inflammatory cytokines (Lull and Block, 2010). It has been suggested that the polarization of microglia between the M1 inflammatory and M2 anti-inflammatory types is important for improving neurological pathology and cognitive decline in Alzheimer’s disease (Sarlus and Heneka, 2017).

The current study is novel, as we evaluated the effects of iso-α-acids on brain inflammation in Alzheimer’s disease after its onset. It was previously demonstrated that a long-term intake of iso-α-acids has a preventive effect against Alzheimer’s pathology in 5 × FAD mice by suppressing inflammation and Aβ deposition in the brain (Ano et al., 2017). In the study, iso-α-acids were fed as a component (0.05% w/w) of the daily diet, so the daily treatment amount was not strictly controlled. In the present study, we evaluated the therapeutic effects of iso-α-acids on 6-month-old 5 × FAD mice displaying Aβ deposition, inflammation, and cognitive impairment. Treatment with iso-α-acids at 1 mg/kg for 7 days suppressed inflammation in the hippocampus and improved hippocampus-dependent object recognition memory (Cohen and Stackman, 2015). Iso-α-acids treatment also reduced the level of soluble Aβ_1-42_ in the hippocampus, which is known to be elevated with inflammation (Lee et al., 2008). Hippocampus-dependent memory is known to decline with inflammation in the hippocampus (Lee et al., 2008), and it has been reported that PPAR-γ activation improves object recognition test performance in 5 × FAD mice (Escribano et al., 2009). These results suggest that suppression of inflammation in the hippocampus by iso-α-acids may contribute to the improvement in memory impairment in Alzheimer’s disease model mice.

At last, to evaluate the effects of iso-α-acids on neural activity, J20 Alzheimer’s disease model mice treated with iso-α-acids at 1 mg/kg for 7 days were subjected to MEMRI analysis after exploration of a novel environment. Our group previously reported that 12-month-old J20 mice displayed hyperactivity detected by MEMRI in hippocampal neurons induced by exploration of a novel environment (Yoshikawa et al., 2018). J20 mice and 5 × FAD mice express human APP with mutations. J20 mice overexpresses human APP with two mutations linked to FAD (the Swedish and Indiana mutations) (Wright et al., 2013), on the other hand, 5 × FAD mice overexpress APP with mutations of Swedish K670N/M671L, Florida I716V and London V717I and PSEN1 with mutation of M146L and L286V (Oakley et al., 2006). 5 × FAD display more severe amyloid pathology including amyloid deposition and inflammation. So in the present study, we evaluate the 6-month-old 5 × FAD and 12-month-old J20 mice. Treatment with iso-α-acids reduced inflammation and soluble Aβ_1-42_ in the hippocampi of J20 mice, which is consistent with the results of the experiments using 5 × FAD mice. Manganese-detected neural activation in the hippocampi of J20 mice was significantly higher than that in age-matched WT mice, whereas the increase was not observed in the hippocampi—especially the CA1 and CA3 region—of J20 mice treated with iso-α-acids. It is reported that hyperactivity in the hippocampal region in individuals with Mild Cognitive Impairment (MCI) is observed by functional MRI (Yassa et al., 2010). It is also reported that hyperactivity in hippocampus lead to cognitive impairment and improvement of the hyperactivity show therapeutic effects on memory impairment (Bakker et al., 2012). It has been also reported that oligomeric Aβ induces neural hyperactivity in hippocampus of Alzheimer’s disease model mice (Busche et al., 2012). In hAPP transgenic mice, Aβ-induced dysfunction of inhibitory interneurons induces aberrant synchrony in neural networks (Palop and Mucke, 2010; Busche et al., 2012). Taken together, it is suggested that suppression of inflammation and the level of soluble Aβ by iso-α-acids administration in Alzheimer’s disease model mice might contribute to the improvement of neural activity in the hippocampus, and resulting memory improvement, especially for the individuals with MCI. On the other hand, the relationship between suppression of hippocampal inflammation and attenuation of neural hyperactivity by iso-α-acids should be investigated in the further study.

Because iso-α-acids are generated from α-acids in hops, which has been used in brewing for more than 1,000 years, they are considered safe for consumption. The amount of iso-α-acids used in the present study was 1 mg/kg, whereas beer generally contains iso-α-acids at 20–50 μg/ml; thus, the amount of iso-α-acids in approximately 1 l of beer might be equivalent to an effective dosage in humans. However, as beer also contains alcohol, it is difficult to calculate the reduced volume. Given the mounting evidence that iso-α-acids may be beneficial for cognition, a clinical trial is needed to further assess their effects in human populations.

## Author Contributions

YA conducted most of biochemical analysis and wrote most of the paper. MY, MM, and AT conducted the experiment using MRI using transgenic mice and analyzed the data, and AT also wrote the manuscript. YT, KU, and HN conducted the experiment using 5 × FAD model mice, and HN also wrote the manuscript.

## Conflict of Interest Statement

YA is employed by Kirin Co., Ltd. The remaining authors declare that the research was conducted in the absence of any commercial or financial relationships that could be construed as a potential conflict of interest.

## Footnotes

1 http://jaxmice.jax.org/strain/006554.html
